# Supplementary material for: Organic radical ferroelectric crystals with martensitic phase transition
Source: Nat Commun. 2023 Sep 20;14:5854. doi: 10.1038/s41467-023-41560-8 (PMC10511434; doi:10.1038/s41467-023-41560-8)
Supplement: Supplementary file 1 — Supplementary Information [file 41467_2023_41560_MOESM1_ESM.pdf]

Supplementary Information

Organic Radical Ferroelectric Crystals with Martensitic Phase Transition

Zhang *et al.*

Supplementary Figures

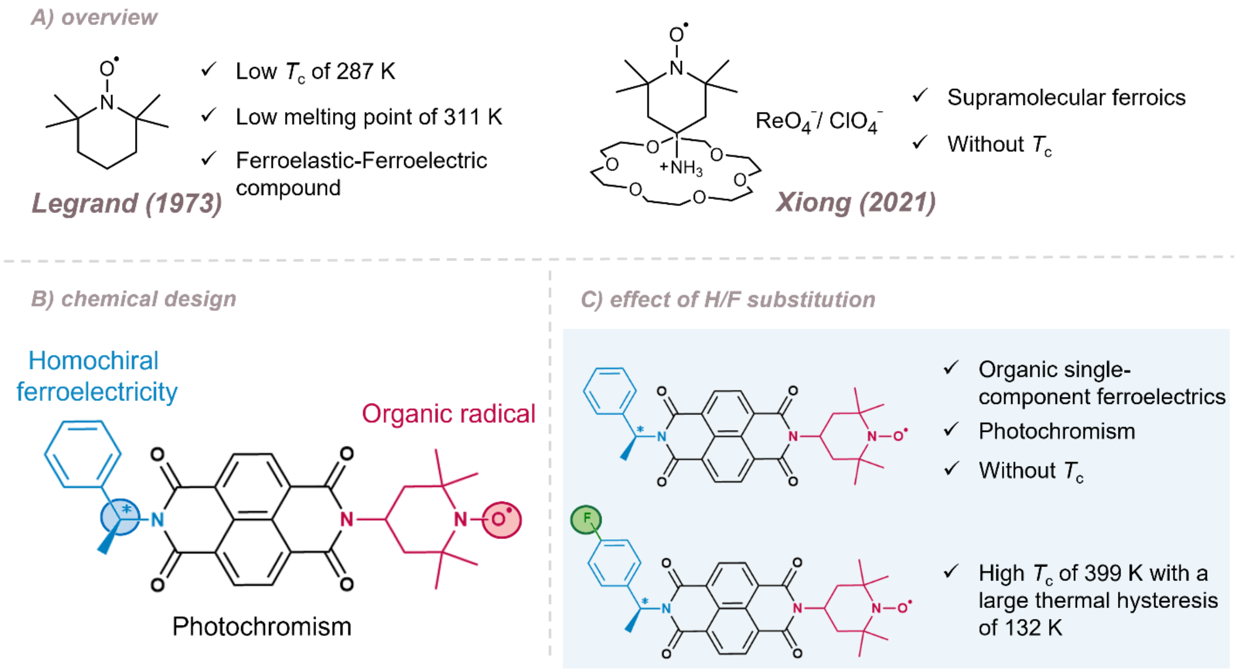

Supplementary Fig. 1: (A) Ferroelectrics with TEMPO radicals. (B) Chemical design strategy. (C) Effects of H/F substitution in NDI derivatives.

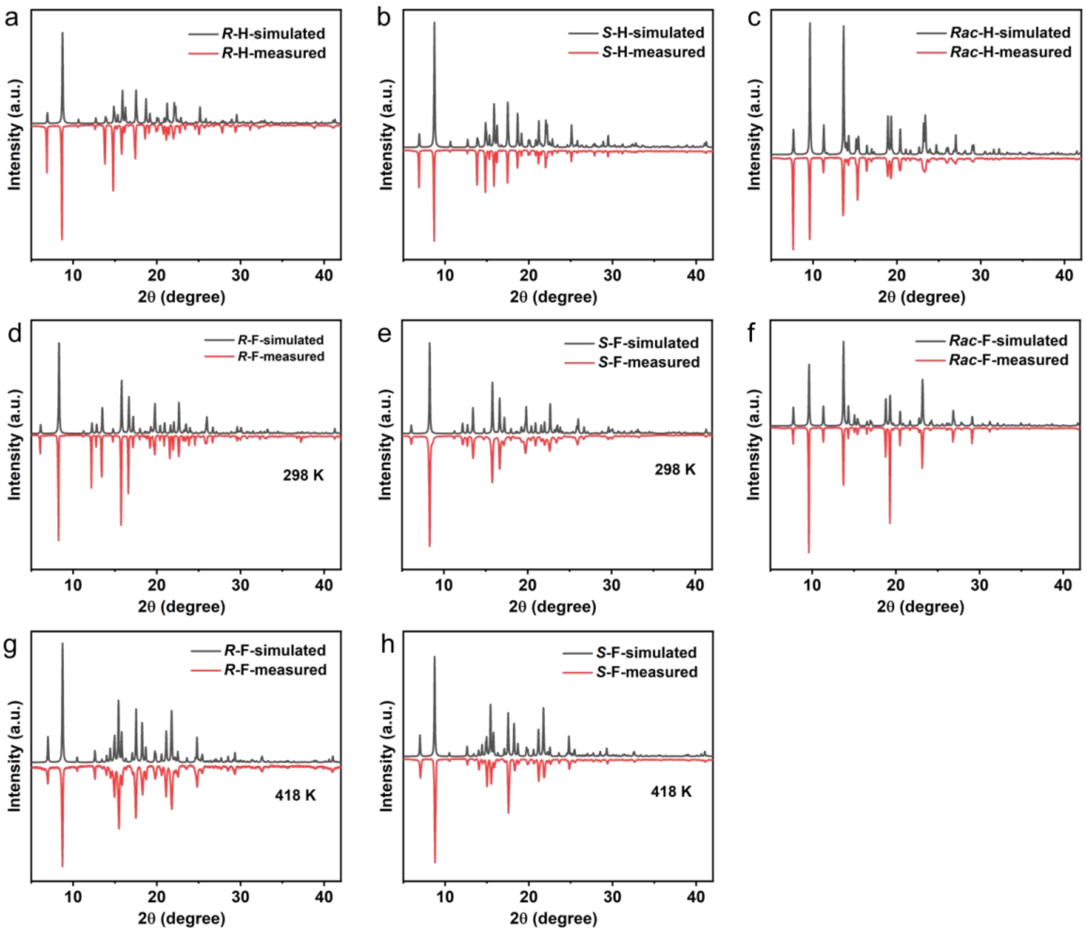

Supplementary Fig. 2: The measured PXRD patterns of *R*-H (a), *S*-H (b), *Rac*-H (c), *R*-F (d), *S*-F (e) and *Rac*-F (f) at 298 K and *R*-F (g) and *S*-F (h) at 418 K match well with the simulated ones from crystallographic data, verifying the good phase purities.

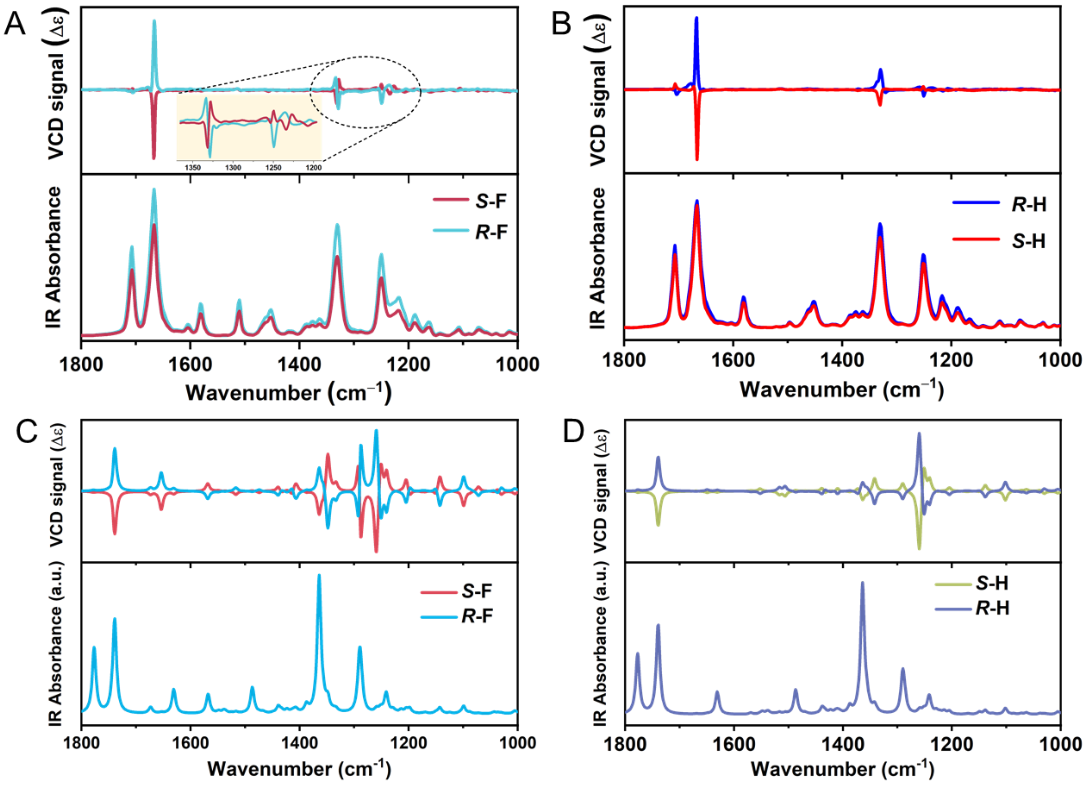

Supplementary Fig. 3: The IR and VCD spectra of *S*-/*R*-F (A) and *S*-/*R*-H (B) and the corresponding calculated ones of *S*-/*R*-F (C) and *S*-/*R*-H (D).

VCD measurement is a powerful tool to detect and characterize chiral compounds in the infrared region. Supplementary Fig. 3A showed the VCD and corresponding IR absorbance spectra of *S*-F and *R*-F. Their VCD spectra exhibited a mirror relationship revealing their enantiomorphic feature. Specifically, there is one pair of strong signals at 1667 cm^{-1} and several relatively weak signals at 1333, 1328 and 1250 cm^{-1}. The strongest VCD signal at 1667 cm^{-1} was assigned to the bending vibration of the C*–C bond induced by the stretching vibration of benzene in the 4-(fluorophenyl)ethyl group. The attribution of the peaks was based on the DFT calculation. After geometric optimization using the B3LYP/6-31G(d) method, the DFT-calculated VCD spectra were consistent with the measured ones. The slight peak shift between the measured and calculated spectra is on account of different molecular conformation. The enantiomers of *S*-/*R*-H also showed similar characteristics.

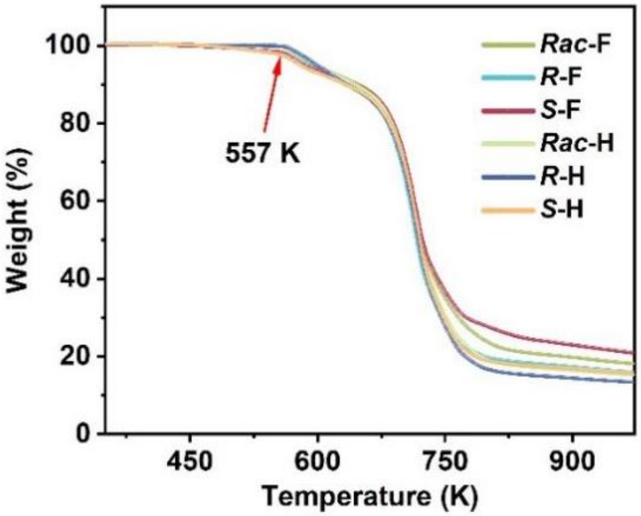

Supplementary Fig. 4: TGA analyses of *R*-F, *S*-F, *Rac*-F, *R*-H, *S*-H, and *Rac*-H.

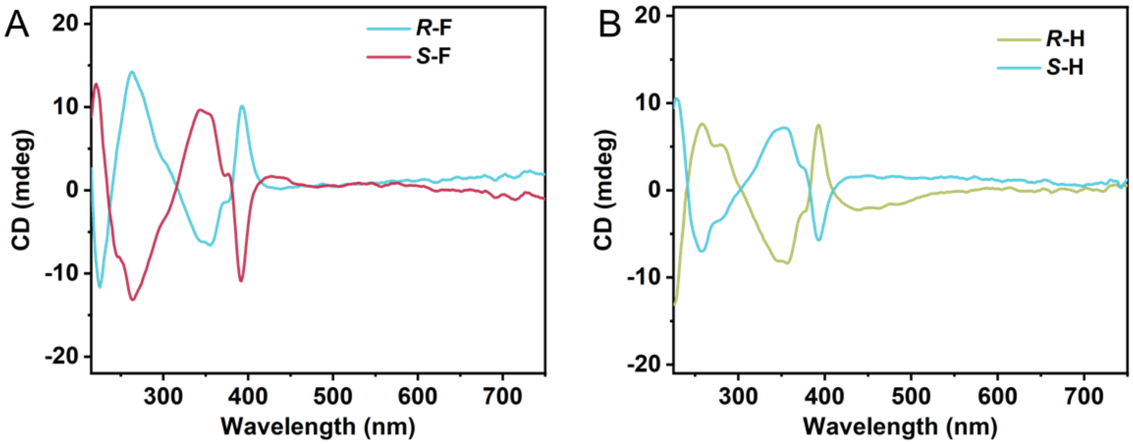

Supplementary Fig. 5: CD spectra of *S*-/*R*-F (A) and *S*-/*R*-H (B).

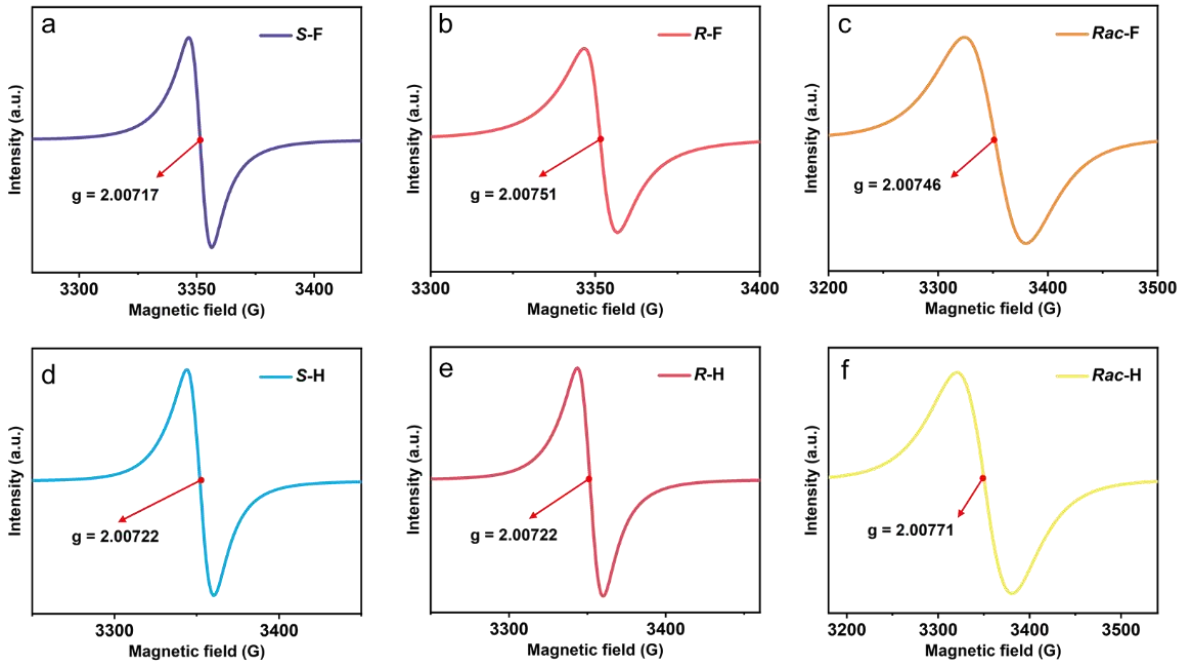

Supplementary Fig. 6: EPR spectra of *S*-F (a), *R*-F (b), *Rac*-F (c), *S*-H (d), *R*-H (e) and *Rac*-H (f) in the solid state (powder).

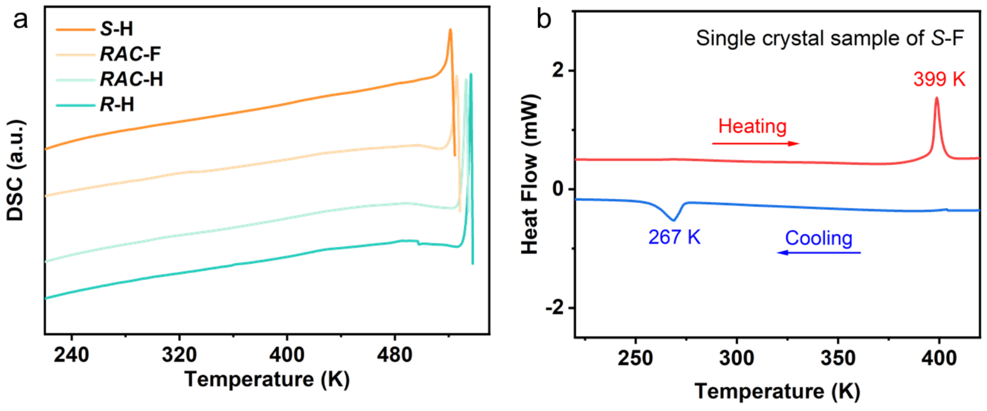

Supplementary Fig. 7: (a) The DSC curves of *Rac*-F and *S*-/*R*-/*Rac*-H powders in the heating mode. (b) The DSC curves of *S*-F single crystal. The DSC results of single crystals and crystalline powder were basically the same except that the peak intensity of the single crystal samples was stronger than that of the powder ones.

On the basis of Boltzmann's equation, the change of entropy (ΔS) could be expressed as follows: $\Delta S = R\ln N$, where R is the ideal gas constant (8.314 J K^{-1} mol^{-1}) and N represents the ratio of the number of possible configurations in HTP and LTP. Taking *S*-F as an example, ΔS is the entropy change at T_c, and $\Delta S_{(S)} = \Delta H / T = 12.13$ J K^{-1} mol^{-1}, where $T = 399$ K, and $\Delta H = 4839.87$ J mol^{-1} (enthalpy change obtained from the DSC measurement). Similarly, $\Delta S_{(R)}$ is equal to 13.56 J K^{-1} mol^{-1}. Thus, the $N_{(S)}$ and $N_{(R)}$ values were deduced to be 4.3 and 5.1, respectively.

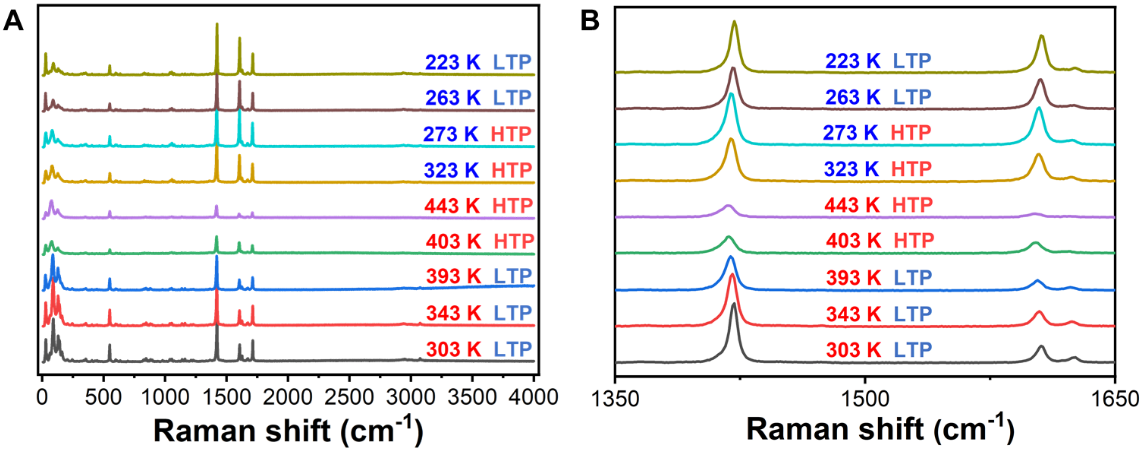

Supplementary Fig. 8: Temperature-dependent Raman spectra of *R*-F at the range of 40–4000 cm^{-1} (A) and at the range of 1350–1650 cm^{-1}(B).

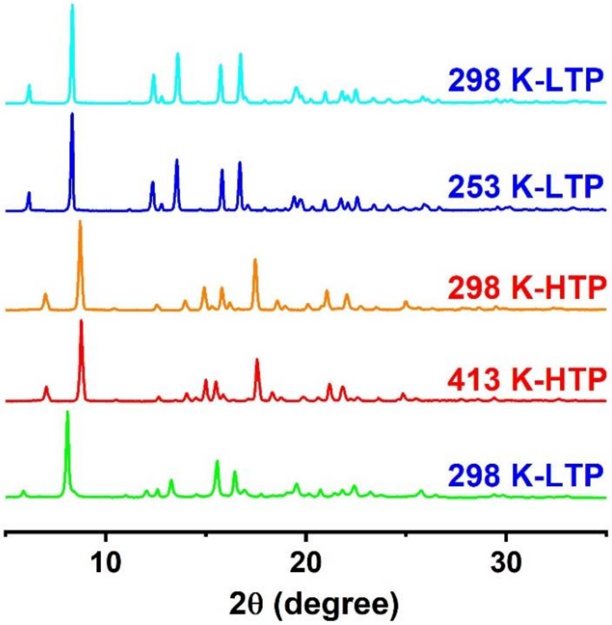

Supplementary Fig. 9: Temperature-dependent PXRD results of *S*-F.

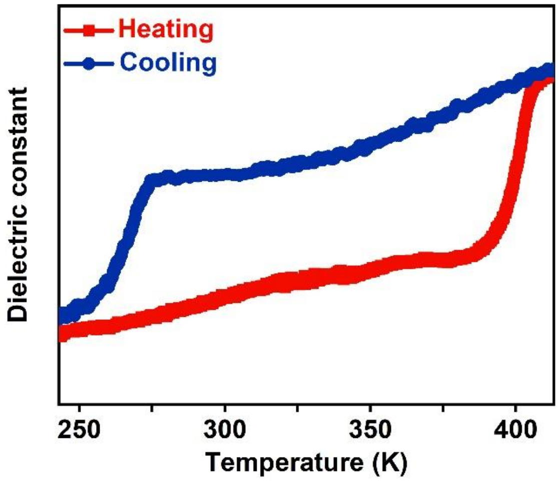

Supplementary Fig. 10: Dielectric constant curves of *R*-F.

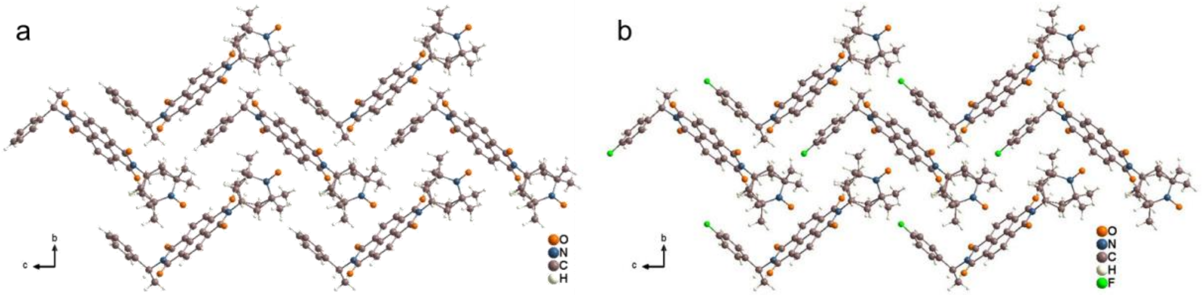

Supplementary Fig. 11: Packing view of *Rac*-H (a) and *Rac*-F (b) along *a*-axis.

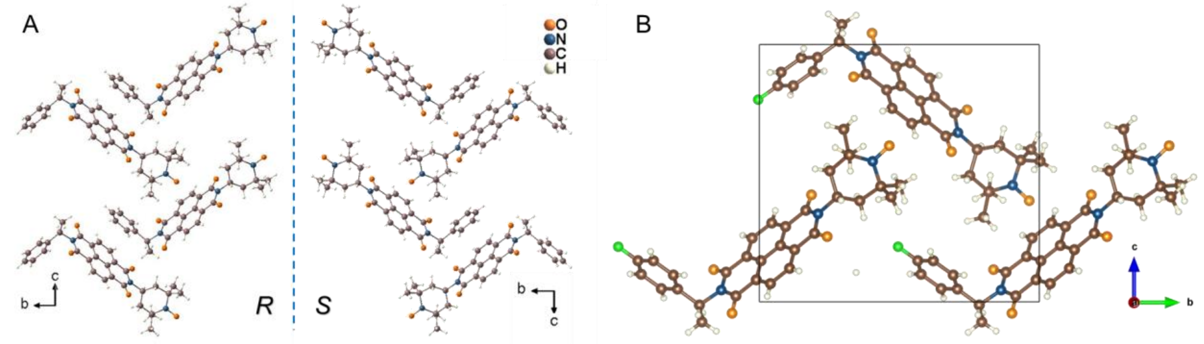

Supplementary Fig. 12: (A) Packing view of *R*-H (left) and *S*-H (right) along *a*-axis showing a mirror relationship. (B) Packing view of *R*-F along *a*-axis.

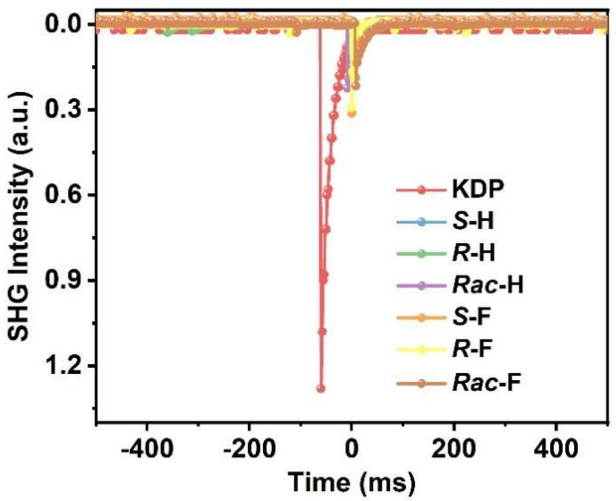

Supplementary Fig. 13: SHG signals of *S*-F, *R*-F, *Rac*-F, *S*-H, *R*-H, *Rac*-H and KDP at room temperature.

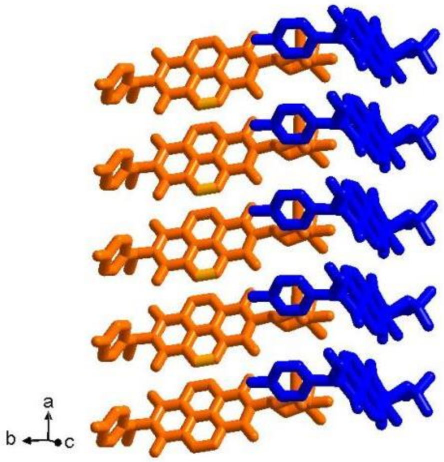

Supplementary Fig. 14: Schematic of columns formed by *S*-/*R*-F packing along *a*-axis.

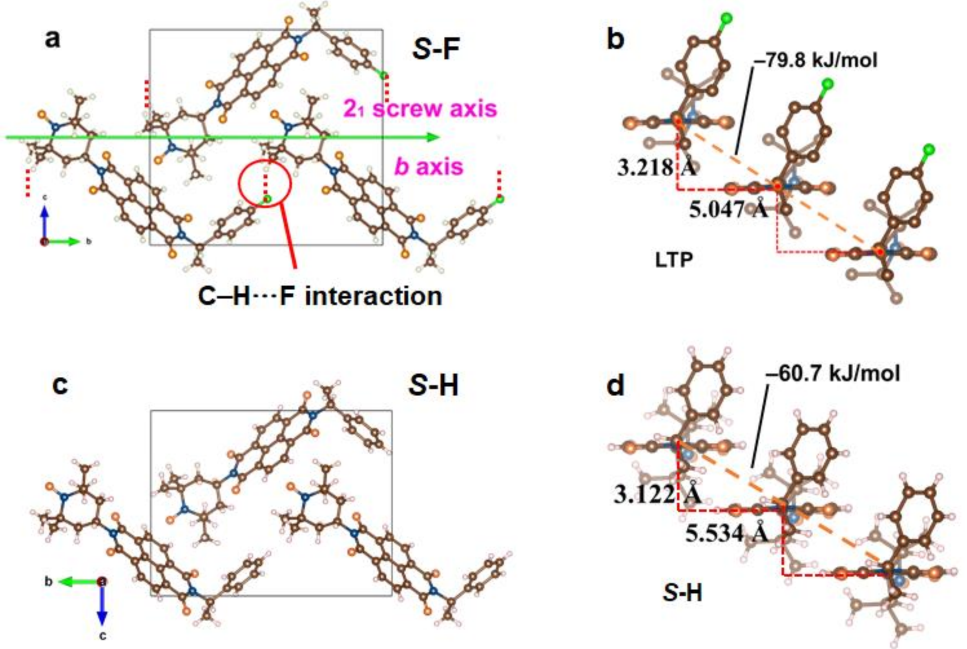

Supplementary Fig. 15: (a) Packing view of *S*-F at LTP along the *a*-axis. Red dashed lines represent C–H···F interaction, which cannot be found in *S*-H. (b) Schematic diagrams of the distance and interaction energy between adjacent *S*-F molecules. H atoms are partly omitted for clarity. (c) Packing view of *S*-H along the *a*-axis. (d) Schematic diagrams of the distance and interaction energy between adjacent *S*-H molecules.

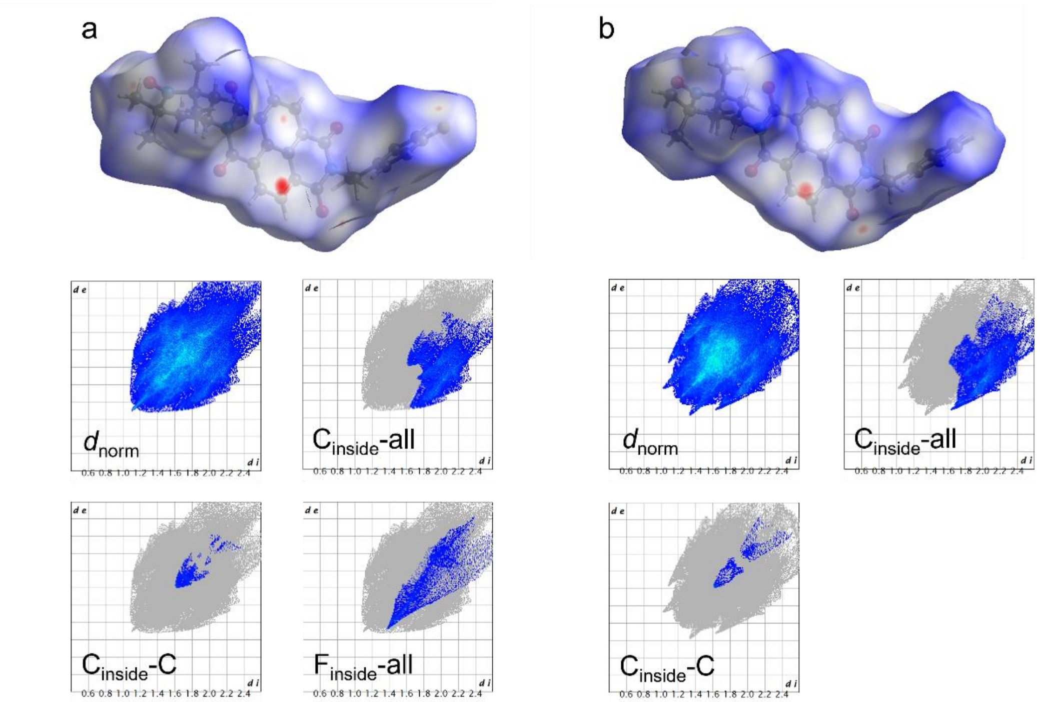

Supplementary Fig. 16: The Hirshfeld d_{norm} surfaces and the 2D fingerprint plots of *S*-F at LTP (a) and *S*-H (b). The interactions between different atoms are marked in the Figures. According to the

red regions shown in Hirshfeld surfaces, *S*-F showed C–H···F interactions as well as the enhanced interlayer π-π interactions in comparison with *S*-H.

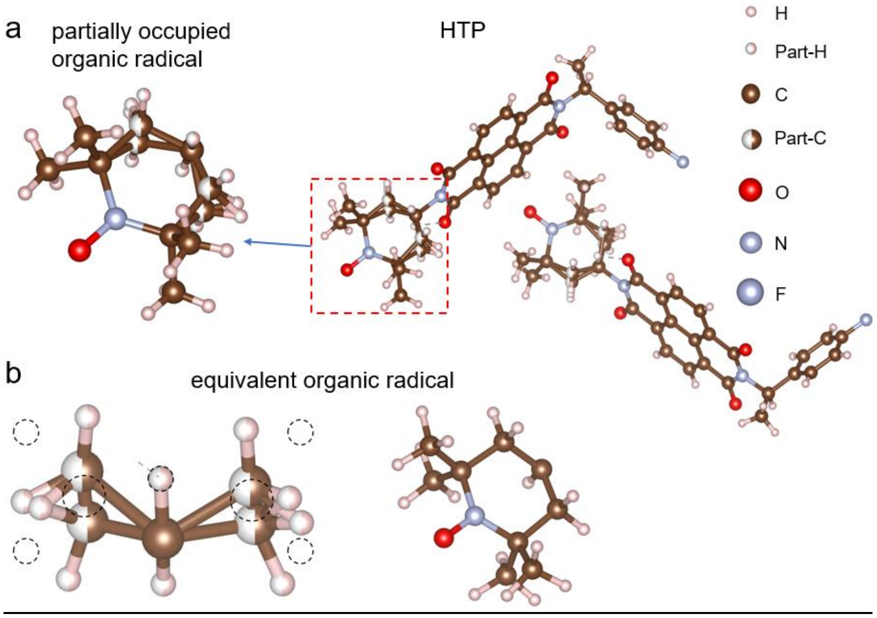

Supplementary Fig. 17: High temperature equivalent structure used in DFT calculation. (a) Right: a HTP unit cell. Left inset: a magnified view of organic radical (in red box). (b) Left: the partially occupied H, C ions and their equivalent positions (dotted circles); Right: the corresponding organic radical without partial occupation.

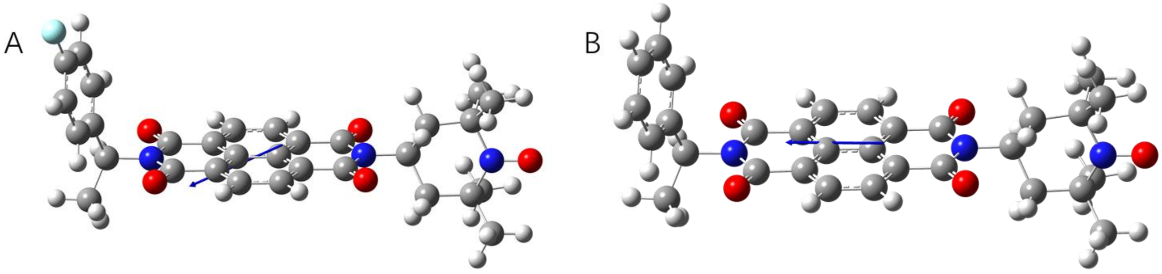

Supplementary Fig. 18: (A) Molecular structure of *S*-F with 2.7804 Debye. The vector components of the molecular dipole on the *a*-, *b*- and *c*-axis are -2.0885, -1.6908 and 0.7143 Debye, respectively. (B) Molecular structure of *S*-H with 2.9959 Debye. The vector components of the molecular dipole on the *a*-, *b*- and *c*-axis are -2.9501, 0.4663 and -0.2348 Debye, respectively.

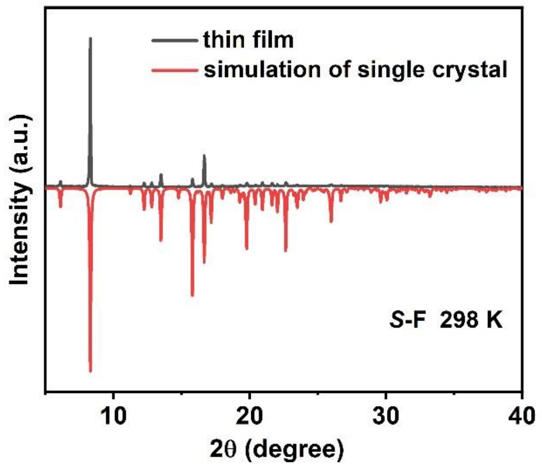

Supplementary Fig. 19: The measured PXRD pattern of the *S*-F thin film matches well with the simulated one derived from the single-crystal structure at 298 K, indicating that the thin film and single crystal of compound *S*-F have the same structure.

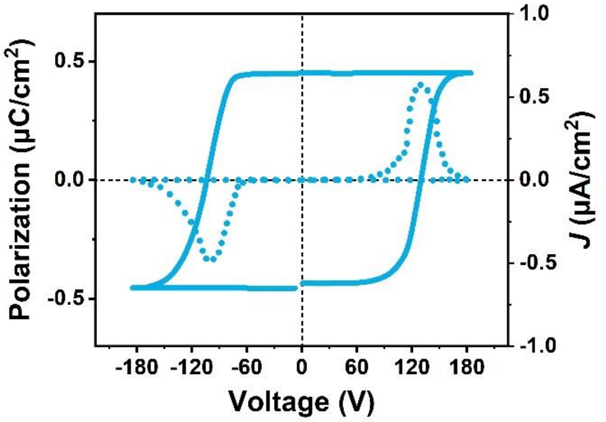

Supplementary Fig. 20: The *J*–*V* (dotted) and *P*–*V* (solid) curves of *R*-F showing typical ferroelectric hysteresis loop.

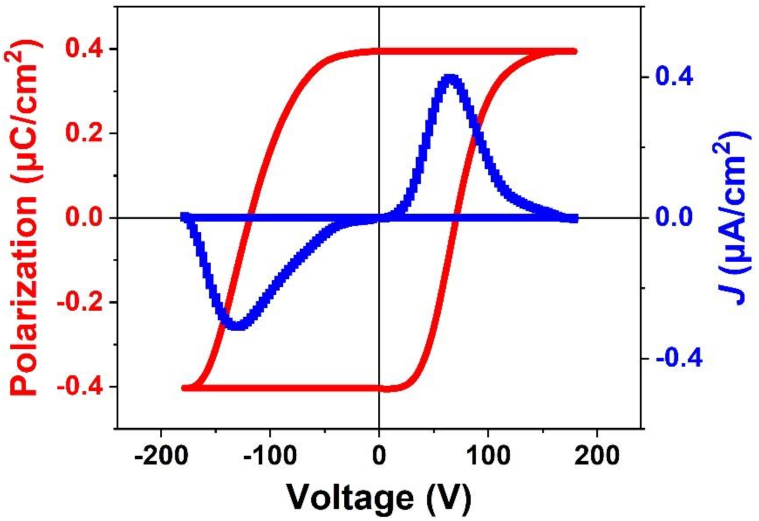

Supplementary Fig. 21: The *J*–*V* (bule) and *P*–*V* (red) curves of *S*-F at HTP measured at room temperature.

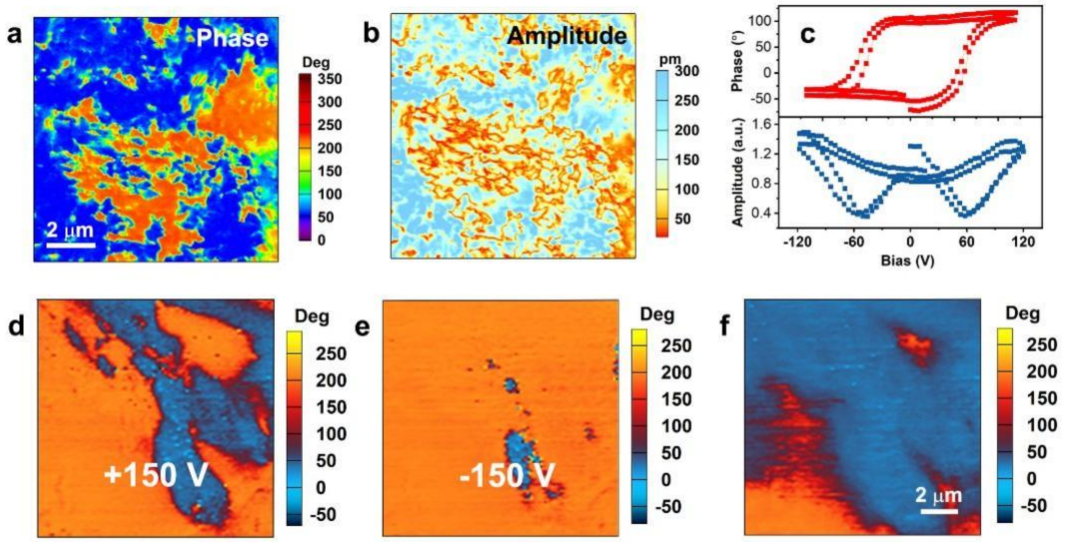

Supplementary Fig. 22: Vertical PFM phase (a) and amplitude (b) images of *R*-F. (c) Phase–voltage hysteresis loop and amplitude–voltage butterfly loop. (d–f) The electrical switching of the ferroelectric domain by applying ±150 V voltage: (d) pristine, (e) after applying a positive bias voltage and (f) after applying a negative bias voltage.

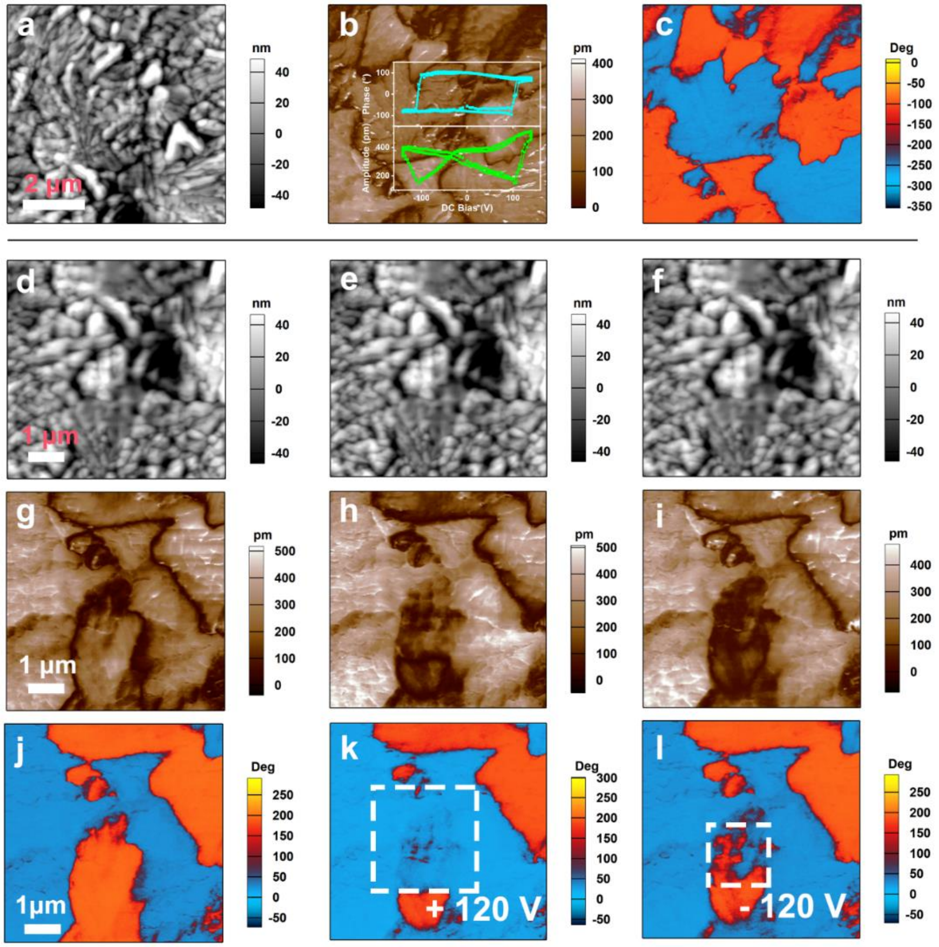

Supplementary Fig. 23: Vertical PFM topography (a), amplitude (b) and phase (c) images of *S*-F in the HTP. (b) Inset: phase–voltage hysteresis loop and amplitude–voltage butterfly loop. Topography (d–f), amplitude (g–i)) and phase (j–l) images of *S*-F before and after applying ±120 V voltage on the boxes.

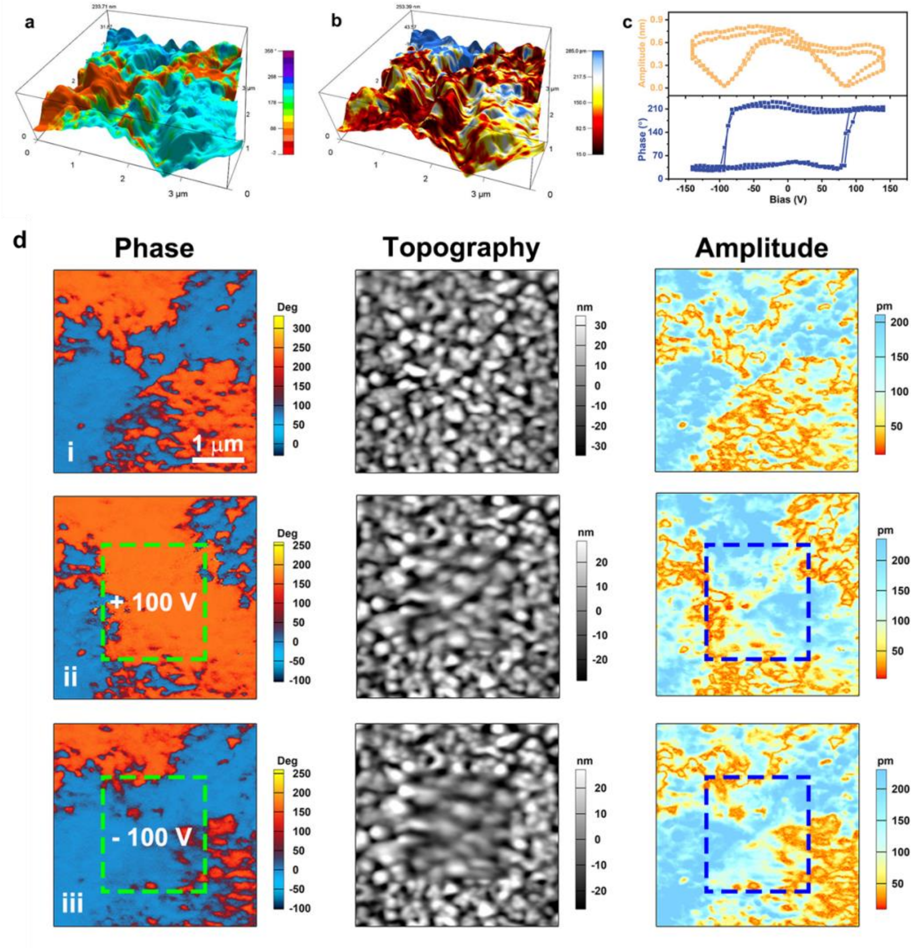

Supplementary Fig. 24: Vertical PFM amplitude (a) and phase (b) images of *S*-H overlaid on the 3D topographic image. (c) Phase–voltage hysteresis loop and amplitude–voltage butterfly loop. (d) The phase, topography and amplitude images of *S*-H before and after applying ±100 V voltage on the boxes.

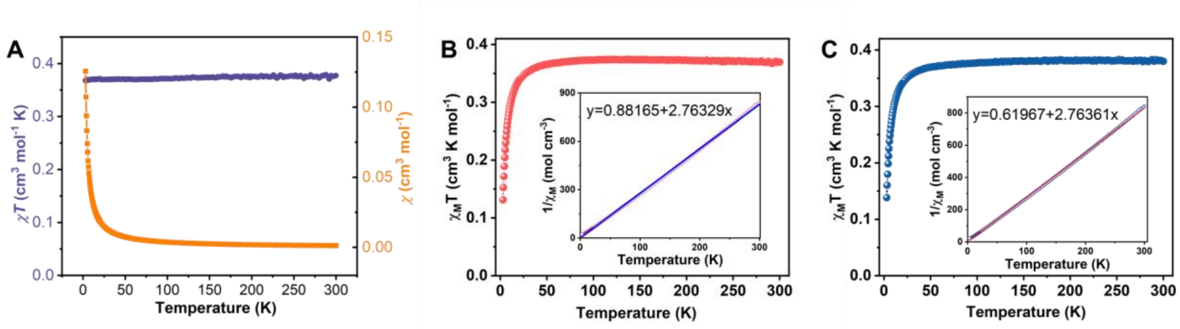

Supplementary Fig. 25: (A) Temperature dependence of $\chi_M T$ and χ for *Rac*-F. Temperature dependence of $\chi_M T$ for *S*-F (B) and *R*-F (C), fitting the Curie–Weiss law (inset).

$$\frac{1}{\chi_M} = \frac{T - \theta}{C}$$

The Curie constants C equal 0.3618/0.3619 cm^3 K mol^{-1} and the fitting Weiss constants θ are –0.22/–0.32 K for *S*- and *R*-F, respectively. This confirms the paramagnetic properties of the enantiomeric compounds.

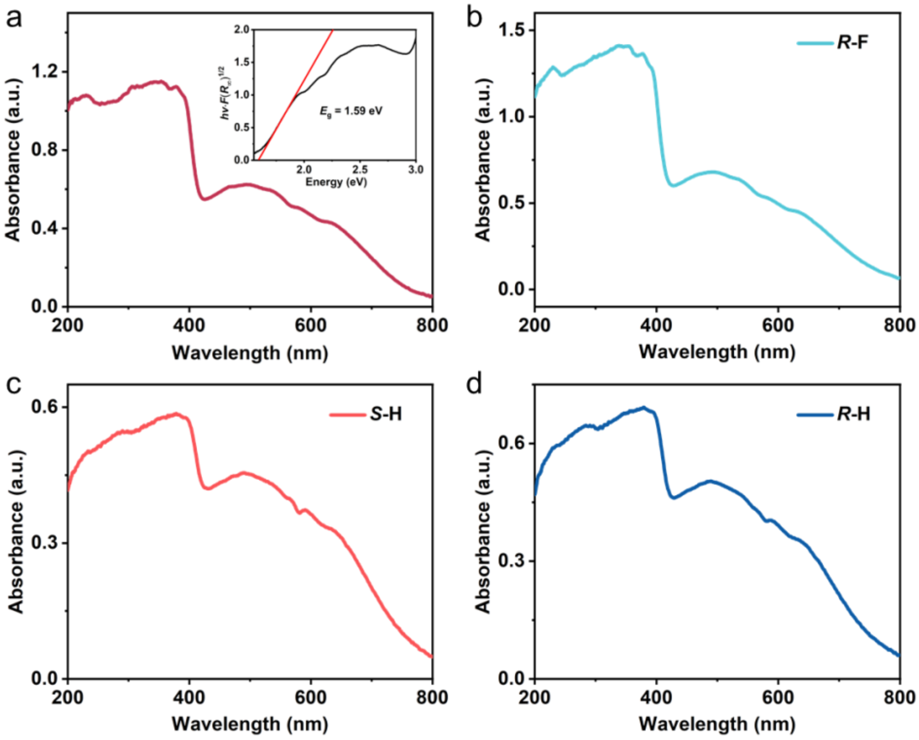

Supplementary Fig. 26: UV-vis absorption spectra of *S*-F (a), *R*-F (b), *S*-H (c) and *R*-H (d) in solid state. (a) Inset: *Tauc* plot. The low-energy broad bands ranging from 425–800 nm could be assigned to the intramolecular charge transfer (ICT) behavior arising from the chiral 4-fluoro-α-methylbenzylamine and TEMPO moieties (donors) to electron-deficient NDI units (acceptor)[7].

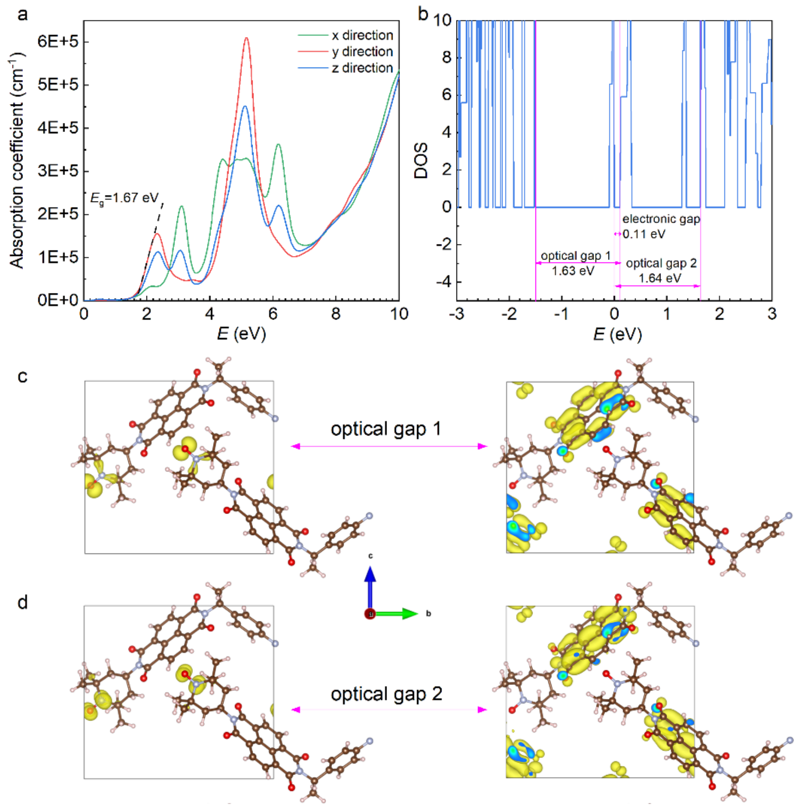

Supplementary Fig. 27: DFT results of optical absorption and electronic structure. (a) Optical absorption coefficient. (b) The density of states. The gaps between valence band maximum and conducting band minimum, as well as two possible optical gaps, are indicated. (c-d) Charge transfer from photon-electron excition. Left: occupied states; Right: excited states. For both two possible optical absorptions, the electron from O–N hybrid state is excited to the neighboring C rings.

For a deeper understanding of the electronic structure, the density of electronic states (DOS) is calculated by DFT. However, the calculated electronic band gap is very narrow (0.11 eV) and contrary to the result acquired from the *Tauc* plot (Supplementary Fig. 25a). This inconsistency might result from a forbidden transition of photons and could be understood through the optical absorption coefficient. The calculated optical absorption band gap (1.67 eV) is consistent with the experimental value and no near-infrared absorption is observed, which indicates that the calculated electronic band gap of 0.11 eV is forbidden. According to the charge density plots, the electrons excited by optical absorption are transferred from O and N ions to neighboring C ions.

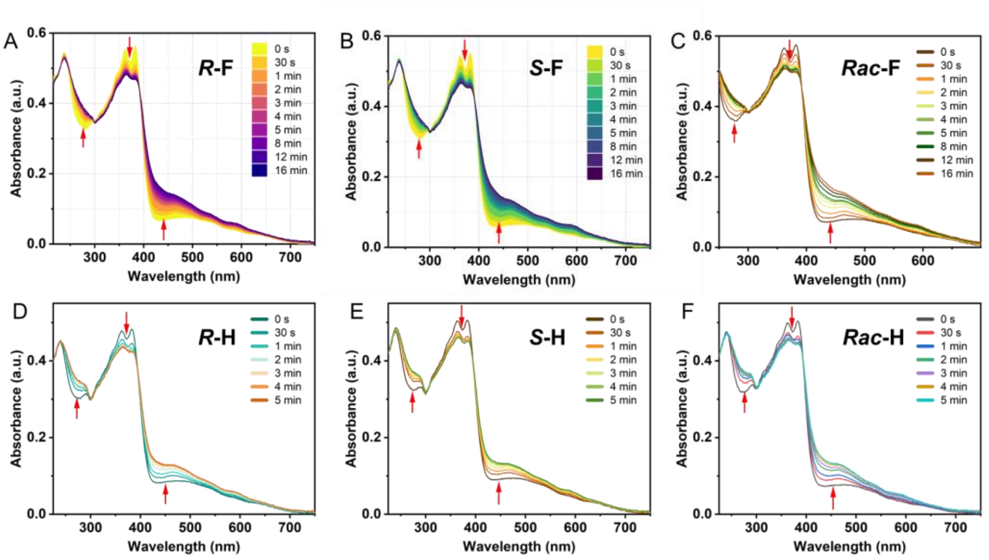

Supplementary Fig. 28: UV/Vis spectra of *R*-F (A), *S*-F (B), *Rac*-F (C), *R*-H (D), *S*-H (E) and *Rac*-H (F) under irradiation of 365 nm light with different time. Taking compound *S*-F as an example, with the time increase of 365 nm light illumination, the absorption intensities at around 280 and 450 nm gradually increased, while the absorption intensities at 362 and 382 nm gradually decreased. This variation reached saturation after light irradiation for 16 minutes. Meanwhile, we can observe apparent photochromic phenomena, where the sample color changed from pale pink to pale yellow. These absorption variations can be assigned to the photoinduced charge transfer between the NDI acceptors and donors, resulting in the generation of NDI radicals, as fully studied previously[7].

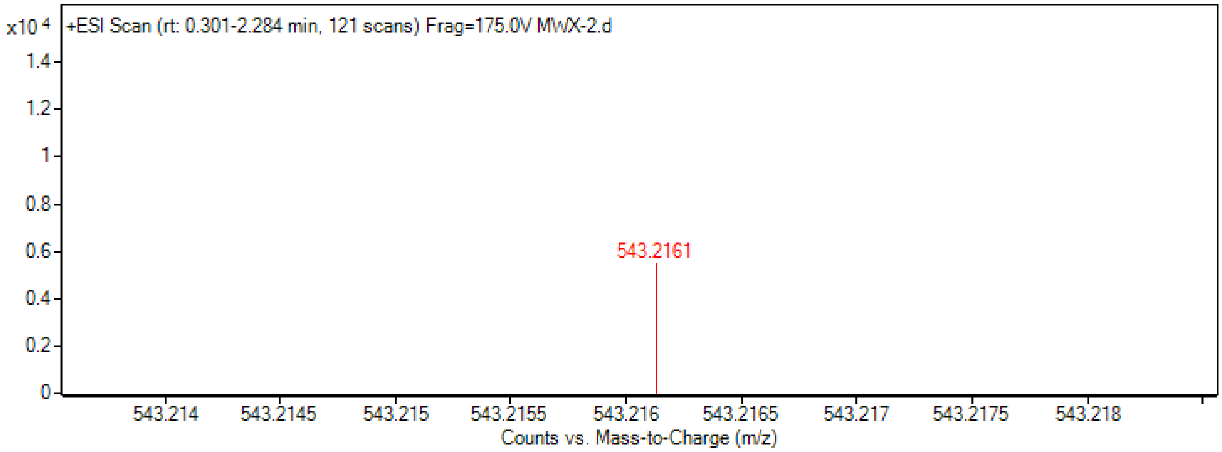

Supplementary Fig. 29: The HRMS spectrum of *S*-F.

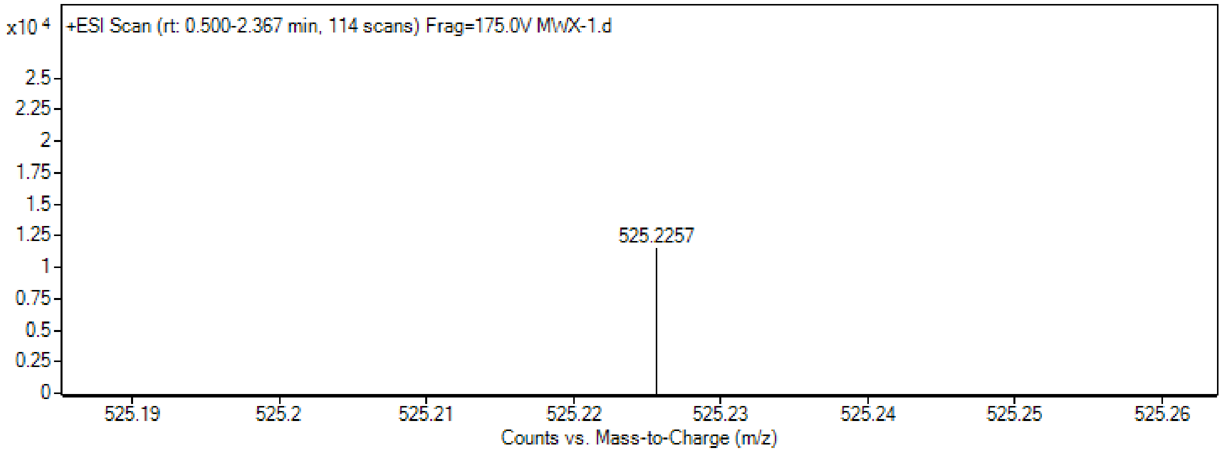

Supplementary Fig. 30: The HRMS spectrum of *S*-H.

Supplementary Tables

Supplementary Table 1: Comparison of P_s, T_c, and ΔT of the reported organic single-component ferroelectrics.

Compound or acronym in the original publication	P_s ($\mu C/cm^2$)	T_c (K)	ΔT (K)	References
***S*- and *R*-F**	**~0.44**	**399**	**132**	**This work**
2-methylbenzimidazole	5–7	-	-	*Crystals* 2021, 11, 1278
N-salicylidene-2,3,4,5,6-pentafluoroaniline	0.84	-	-	*Adv. Sci.* 2021, 8, 2102614
(*S*)-*N*-3,5-di-*tert*-butylsalicylidene-1–4-bromophenylethylamine	-	242<br>336	5<br>4	*J. Am. Chem. Soc.* 2021, 143, 21685–21693
salicylideneaniline	1.95	-	-	*Chem. Eur. J.* 2021, 27, 14831
3,4,5-trifluoro-*N*-(3,5-di-*tert*-butylsalicylidene)aniline	0.87	-	-	*J. Am. Chem. Soc.* 2021, 143, 13816–13823
2-(hydroxymethyl)-2-nitro-1,3-propanediol	8.4	345<br>362	15<br>27	*J. Am. Chem. Soc.* 2020, 142, 13989–13995
nopinic acid		161	4	*Appl. Mater. Today* 2020, 20, 100687
2-(*p*-tolyl)-1*H*-phenanthro[9,10-*d*]imidazole	3	521	-	*Chem. Commun.* 2019, 55, 9610–9613
(*R*)-3-quinuclidinol<br>(*S*)-3-quinuclidinol	6.96(*R*)<br>6.72(*S*)	400 (*R*)<br>398 (*S*)	-	*Proc. Natl. Acad. Sci. U. S. A.* 2019, 116, 13, 5878–5885
4-(4-(methylthio)phenyl)-2,6-di(1*H*-pyrazol-1-yl)pyridine	0.715	-	-	*J. Mater. Chem. C* 2018, 6, 9330–9335
6TP	-	301<br>505	-	*ChemistrySelect* 2018, 3, 10608
5,6-dichloro-2-methylbenzimidazole	-	399	~30	*Nat. Commun.* 2012, 3, 1308
croconic acid	21	-	-	*Nature* 2010, 463, 789–792
2-phenylmalondialdehyde	9	363	-	*Adv. Mater.* 2011, 23, 2098–2103
3-HPLN	3	-	-	
CBDC	2.9	400	-	
1,3,5-trimethylnitrobenzene	2.0	152<br>161	-	*CrystEngComm* 2021, 23, 4005–4012
β-sitosteryl 4-iodocinnamate	4	342	14	*Nat. Commun.* 2022, 13, 6150
tetrakis(4-fluorophenylethynyl)silane	~0.1	475	-	*JACS Au* 2023, 3, 2, 603–609
(*S,S*)-4,4'-(3,3,4,4,5,5-hexafluorocyclopent-1-ene-1,2-diyl)bis[5-methyl-*N*-(1-phenylethyl)thiophene-2-carboxamide]	1.49	-	-	*J. Am. Chem. Soc.* 2022, 144, 19, 8633–8640

orth-I-OA	3.43	-	-	*Adv. Sci.* 2022, 9, 2201702
(7*aR*,10*R*,11*aS*)-12,12-dimethyl-6,6-dioxo-3,4,9,10-tetrahydro-7*H*-7*a*,10-methano-2*H*-1,3-oxazino[2,3-*i*][2,1]benzisothiazol-11(8*H*)-one	2.2	460	-	*Chem. Commun.* 2022, 58, 10361–10364
(*R*, *R*)-(*E*, E)-**1**<br>(*S*, *S*)-(*E*, *E*)-**1**	0.16<br>0.17	343	40	*Angew. Chem. Int. Ed.* 2022, 61, e202200135
2-amino-2′,4,4′,6,6′-pentafluoroazobenzene	1.83	433	-	*Chem. Sci.* 2022, 13, 4936–4943

Supplementary Table 2: Molecular ferroelectrics with large thermal hysteresis.

**Compound or acronym in the original publication**	**Δ*T***	**References**
diisopropylammonium bromide	8 K	*Science* 2013, 339, 425–428
D-*chiro*-inositol-$SiMe_3$	15 K	*Angew. Chem. Int. Ed.* 2022, 61, e202210809
TMCM-$MnCl_3$	~18 K	*Science* 2017, 357, 306–309
[3-oxoquinuclidinium]ClO_4	35 K	*J. Am. Chem. Soc.* 2019, 141, 1781–1787
MDABCO-NH_4I_3	~56 K	*Science* 2018, 361, 151–155
[3.2.1-dabco]BF_4	70 K	*J. Am. Chem. Soc.* 2020, 142, 1995–2000

Supplementary Table 3: Crystallographic data of *S*-F, *R*-F and *Rac*-F.

**Compound**	***S*-F**		***R*-F**		***Rac*-F**
**Formula**	$C_{31}H_{29}FN_3O_5$		$C_{31}H_{29}FN_3O_5$		$C_{31}H_{29}FN_3O_5$
**Temperature**	298K	418K	298K	418K	298K
**Weight**	542.57		542.57		542.57
**System**	monoclinic	monoclinic	monoclinic	monoclinic	monoclinic
**Space group**	$P2_1$	$P2_1$	$P2_1$	$P2_1$	Pc
a **(Å)**	5.98590(10)	6.6000(3)	5.9937(2)	6.5908(6)	7.8667(2)
b **(Å)**	15.7126(4)	16.8407(7)	15.7196(4)	16.8807(14)	11.4087(2)
c **(Å)**	14.4821(4)	12.6531(6)	14.4474(4)	12.6428(9)	15.6183(3)
α **(°)**	90	90	90	90	90
β **(°)**	92.349(2)	92.536(4)	92.113(2)	92.403(7)	97.225(2)
γ **(°)**	90	90	90	90	90
**V ($Å^3$)**	1360.96(6)	1405.00(11)	1360.29(7)	1405.4(2)	1390.59(5)
**Z**	2	2	2	2	2
**R_1**	0.0582	0.0761	0.0465	0.0974	0.0510
**wR_2**	0.1713	0.2206	0.1326	0.2506	0.1437
**GOF**	1.060	1.025	1.041	1.075	1.045

Supplementary Table 4: Crystallographic data of *S*-H, *R*-H and *Rac*-H.

**Compound**	***S*-H**	***R*-H**	***Rac*-H**
**Formula**		$C_{31}H_{30}N_3O_5$	
**Temperature**		298K	
**Weight**		524.58	
**System**	monoclinic	monoclinic	monoclinic
**Space group**	$P2_1$	$P2_1$	Pc
a **(Å)**	6.3539(2)	6.3336(8)	7.9128(2)
b **(Å)**	16.6303(6)	16.6076(15)	11.5453(2)
c **(Å)**	12.8038(4)	12.7741(11)	15.2940(4)
α **(°)**	90	90	90
β **(°)**	92.557(3)	92.561(9)	97.513(2)
γ **(°)**	90	90	90
**V (Å^3)**	1351.60(8)	1342.3(2)	1385.20(6)
**Z**	2	2	2
**R_1**	0.0566	0.0545	0.0484
**wR_2**	0.1608	0.1440	0.1360
**GOF**	1.016	0.990	1.029

Supplementary Table 5: Temperature-dependent change of the three axes of the crystallographic axes of *S*-F.

**Temperature / K**	***a*** **/ Å**	**sd (*a*)[a]**	***b*** **/ Å**	**sd (*b*)[a]**	***c*** **/ Å**	**sd (*c*)[a]**
300	5.986	0.006	15.79	0.02	14.545	0.018
330	6.008	0.006	15.74	0.02	14.487	0.015
360	6.028	0.005	15.731	0.017	14.48	0.017
390	6.097	0.006	15.86	0.02	14.33	0.02
420	6.607	0.009	16.81	0.02	12.687	0.017
450	6.65	0.02	16.88	0.05	12.79	0.04
420	6.63	0.02	16.89	0.05	12.59	0.04
390	6.53	0.03	16.96	0.07	12.66	0.05
360	6.53	0.03	16.98	0.07	12.63	0.05
330	6.46	0.04	16.89	0.1	12.86	0.07
300	6.44	0.03	17.04	0.08	12.69	0.07
270	6.15	0.02	15.86	0.06	14.21	0.04
240	6.04	0.02	15.72	0.05	14.37	0.04
270	6.1	0.02	15.79	0.05	14.39	0.04
300	6.02	0.02	15.87	0.05	14.34	0.05

[a] Standard deviation

Supplementary Table 6: Optimized lattice parameters of *S*-/*R*-F and *S*-/*R*-H by DFT (Calc.), in comparison with the experimental results (Expt.). The *a*, *b*, and *c* are in units of Å.

S/*R*-F	LTP Calc.	LTP Expt.	HTP Calc.	HTP Expt.
a	5.787	5.986	6.129	6.591
b	15.704	15.713	16.965	16.881
c	14.381	14.482	12.705	12.643
S/*R*-H	LTP Calc.	LTP Expt.		
a	6.087	6.334		
b	16.635	16.608		
c	12.861	12.774		
