# Supplementary material for: Organic radical ferroelectric crystals with martensitic phase transition
Source: Nat Commun. 2023 Sep 20;14:5854. doi: 10.1038/s41467-023-41560-8 (PMC10511434; doi:10.1038/s41467-023-41560-8)
Supplement: Supplementary file 3 — Description of additional supplementary files [file 41467_2023_41560_MOESM3_ESM.pdf]

Description of additional supplementary files

Supplementary Movie 1: The ʃumping crystal’ phenomenon of the fluorinated compounds observed under microscope.

Supplementary Movie 2: An animation of the HTP-LTP transition trace in our CI-NEB simulation.
